# Effectiveness and Safety of Remdesivir for the Treatment of COVID-19 Patients with Liver Cirrhosis: A Retrospective Cohort Study

**DOI:** 10.3390/life15040512

**Published:** 2025-03-21

**Authors:** Yi-Ching Wong, Chip-Jin Ng, Yan-Bo Huang, Shou-Yen Chen

**Affiliations:** 1 Department of Emergency Medicine, Chang Gung Memorial Hospital, College of Medicine, Chang Gung University, Taoyuan 333, Taiwan; gwen8223@gmail.com (Y.-C.W.); ngowl@ms3.hinet.net (C.-J.N.); yanhusuo79619@gmail.com (Y.-B.H.); 2 Graduate Institute of Management, College of Management, Chang Gung University, Taoyuan 333, Taiwan

**Keywords:** COVID-19, liver cirrhosis, remdesivir, liver function, safety, efficacy

## Abstract

Background: Patients with liver cirrhosis are at an increased risk of mortality from coronavirus disease 2019 (COVID-19). Remdesivir, an adenosine analog, exhibits activity against severe acute respiratory syndrome coronavirus 2 (SARS-CoV-2) and is thus recommended for inpatients with COVID-19. This study evaluated the effectiveness and safety of remdesivir in patients with COVID-19 and liver cirrhosis. Methods: This retrospective study was conducted using data from Taiwan’s largest healthcare system. The study cohort comprised adult patients with COVID-19 and liver cirrhosis who visited our emergency department between April 2021 and September 2022. Remdesivir’s adverse effects, including bradycardia, anemia, unstable glucose levels, and abnormal liver function test results, were recorded. Treatment outcomes were assessed in terms of hospitalization duration, mortality, intubation, and intensive care unit admission. Results: This study included 1368 patients with COVID-19 and liver cirrhosis, of whom 46 received remdesivir. Remdesivir recipients were older (66.5 vs. 62 years; *p* = 0.042) and had a higher rate of oxygen therapy use (56.52% vs. 32.22%; *p* = 0.001) than nonrecipients. Common adverse effects of remdesivir included lower heart rates (83 vs. 96 bpm; *p* < 0.001) and decreased hemoglobin levels (9.5 vs. 10.2 g/dL; *p* = 0.003) without fatal consequences. No statistically significant difference between remdesivir recipients and nonrecipients in hospitalization duration, intubation rates, or mortality rates was found. Conclusions: Remdesivir is safe for treating COVID-19 in patients with liver cirrhosis. Although remdesivir recipients exhibited trends toward improved outcomes in our study, large-scale studies are required to confirm its efficacy in this population.

## 1. Introduction

Coronavirus disease 2019 (COVID-19) is an infectious disease caused by the severe acute respiratory syndrome coronavirus 2 (SARS-CoV-2), which was first identified in Wuhan, China in December 2019 [1]. It rapidly induced a global pandemic that was claimed by the World Health Organization (WHO) on 11 March 2020 [2]. Older adults and individuals with underlying conditions such as cardiovascular disease, diabetes, chronic respiratory diseases, chronic kidney disease, chronic liver disease, immunocompromised state, or cancer had more severe illness and higher mortality with COVID-19 [3].

Patients with liver cirrhosis face an increased risk of mortality from COVID-19 [4,5,6]. Studies have shown elevated rates of ICU admission, the need for renal replacement therapy, and mortality in COVID-19 correlating with the severity of liver disease [6,7]. Acute hepatic decompensation occurred in 24–46% of patients with COVID-19, with 23–28% developing acute-on-chronic liver failure [7,8]. Decompensation events included new or worsening ascites, worsening jaundice, hepatic encephalopathy, spontaneous bacterial peritonitis, and variceal hemorrhage [7,8]. Respiratory failure was reported as the primary cause of death, followed by hepatic and cardiac causes [6,9,10]. The mortality rate associated with the original strain increased to 32–34% in patients with cirrhosis, compared to 8–12% in those with chronic liver disease but without cirrhosis. [6,7,9,11,12].

Remdesivir is an adenosine analog that binds to the viral RNA-dependent RNA polymerase, causing premature termination of RNA transcription and subsequent inhibition of viral replication. This drug has been demonstrated to exhibit in vitro and in vivo activity against severe acute respiratory syndrome coronavirus 2 (SARS-CoV-2) [13]. The National Institutes of Health (NIH) recommends remdesivir for inpatients with a high risk of severe disease who do not require oxygen therapy and for those requiring conventional oxygen therapy [14,15,16]. Similar recommendations have been made by the Infectious Diseases Society of America and the World Health Organization (WHO) [17,18,19].

In accordance with the reimbursement conditions of Taiwan’s National Health Insurance system, remdesivir is prescribed only to patients with severe COVID-19, particularly those requiring oxygen therapy or presenting with radiologic evidence of pneumonia on chest X-rays. The recommended treatment protocol involves a 5-day regimen of remdesivir, ideally administered concurrently with dexamethasone [20].

Although commonly prescribed for COVID-19 patients, no consistent benefit has been observed in randomized controlled trials of remdesivir [16,21,22,23,24,25,26,27,28,29,30]. Among patients with non-severe disease without hypoxia or oxygen therapy requirement, remdesivir recipients had a reduced hospitalization duration and improved clinical outcomes comparing to nonrecipients, but the results were nonsignificant [28,31]. For patients with severe disease, some studies have reported varied benefits of remdesivir, including shortened time to recovery, reduced duration of hospitalization, prevention of disease progression, reduced rate of intubation, and reduced mortality rates [16,22,27,28,29].

Even though remdesivir is recommended for patients with COVID-19, this drug has some adverse effects. Common adverse effects include nausea, reduced hemoglobin levels, reduced glomerular filtration rate, increased blood creatinine levels, increased serum glucose levels, abnormal liver function test results, and bradycardia [16,32,33,34].

The US Food and Drug Administration approved the use of remdesivir for treating COVID-19 in patients with mild, moderate, or severe hepatic decompensation on 24 August 2023 [35]. This decision was based on the findings of a phase 1 study on the safety and pharmacokinetics of this drug that included 16 patients with moderate to severe hepatic decompensation (GS-US-540-9014) and 16 matched individuals with normal liver function [36]. However, few studies have focused on the use of remdesivir for treating COVID-19 in patients with liver cirrhosis. This study aims to bridge the knowledge gap by evaluating the safety and effectiveness of remdesivir in COVID-19 patients with liver cirrhosis.

## 2. Methods

### 2.1. Study Design and Data Source

This retrospective study was conducted using data from the Chang Gung Research Database (CGRD) [37]. The CGRD contains deidentified data from the electronic health records of Chang Gung Memorial Hospital (CGMH), Taiwan’s largest hospital system. The database undergoes systematic annual updates to incorporate new data from CGMH. CGMH has ten branches across Taiwan. With a capacity of 10,070 beds, the hospital admits more than 280,000 patients annually. In 2023, the numbers of outpatient and emergency department (ED) visits at CGMH exceeded 9,400,000 and 580,000, respectively. This study was approved by the Institutional Review Board of CGMH (permit number: 202301643B0) and informed consent of participation was waived.

### 2.2. Study Setting and Population

From the CGRD, we collected data of patients with COVID-19 who had visited our emergency department between April 2021 and September 2022. All COVID-19 diagnoses were confirmed through polymerase chain reaction (PCR) testing. Patients with liver cirrhosis were identified based on the *International Classification of Diseases, Tenth Revision, Clinical Modification* (ICD-10-CM) codes for cirrhosis recorded in the outpatient setting within the three months prior to their ED visits. The ICD-10-CM codes used were as follows: K703, K7030, K7031, K717, K74, K743, K744, K745, K746, K7460, K7469, and P7881. We excluded patients without liver cirrhosis, those under 18 years of age, those not requiring hospitalization, and those with a “do not resuscitate (DNR)” order. The basic demographic data included information on age, sex, initial vital signs, laboratory results, and underlying medical conditions. The model for end-stage liver disease (MELD) and WHO ordinal severity scale scores were calculated; in addition, the WHO inflammation risk categories were explored [38,39,40]. We developed a 5-point scale based on radiographic lung images to assess disease severity based on radiologists’ reports. Direct radiographic image evaluation was not possible because our database was de-identified and the images were unavailable. We analyzed the treatment regimens and outcomes—that is, including length of hospital stay (LOS), mortality rate, intubation rate, and intensive care unit (ICU) admission. Patients were stratified into two groups on the basis of remdesivir use.

### 2.3. Outcome Assessment

To evaluate the safety of remdesivir in patients with liver cirrhosis, we compared the presence of common side effects of the drug, such as bradycardia, anemia, unstable glucose levels, and abnormal liver function test results, between remdesivir recipients and nonrecipients.

To evaluate the effectiveness of remdesivir in patients with liver cirrhosis, we compared various treatment outcomes, including LOS, intubation rates, ICU admission, and hospital mortality rates, between remdesivir recipients and nonrecipients.

### 2.4. Statistical Analysis

Data were analyzed using SPSS (version 24.0; IBM Corporation, Armonk, NY, USA) for Windows. Continuous data were compared using the Student *t* and Welch *t* tests, whereas categorical data were compared using the Pearson chi-square test. To mitigate potential bias resulting from interpersonal differences, we performed one-to-one propensity score matching to match remdesivir recipients with nonrecipients. Our logistic regression model was adjusted for the following covariates: age, sex, and Charlson Comorbidity Index score. Significance was set at *p* < 0.05.

## 3. Results

**Figure 1:**
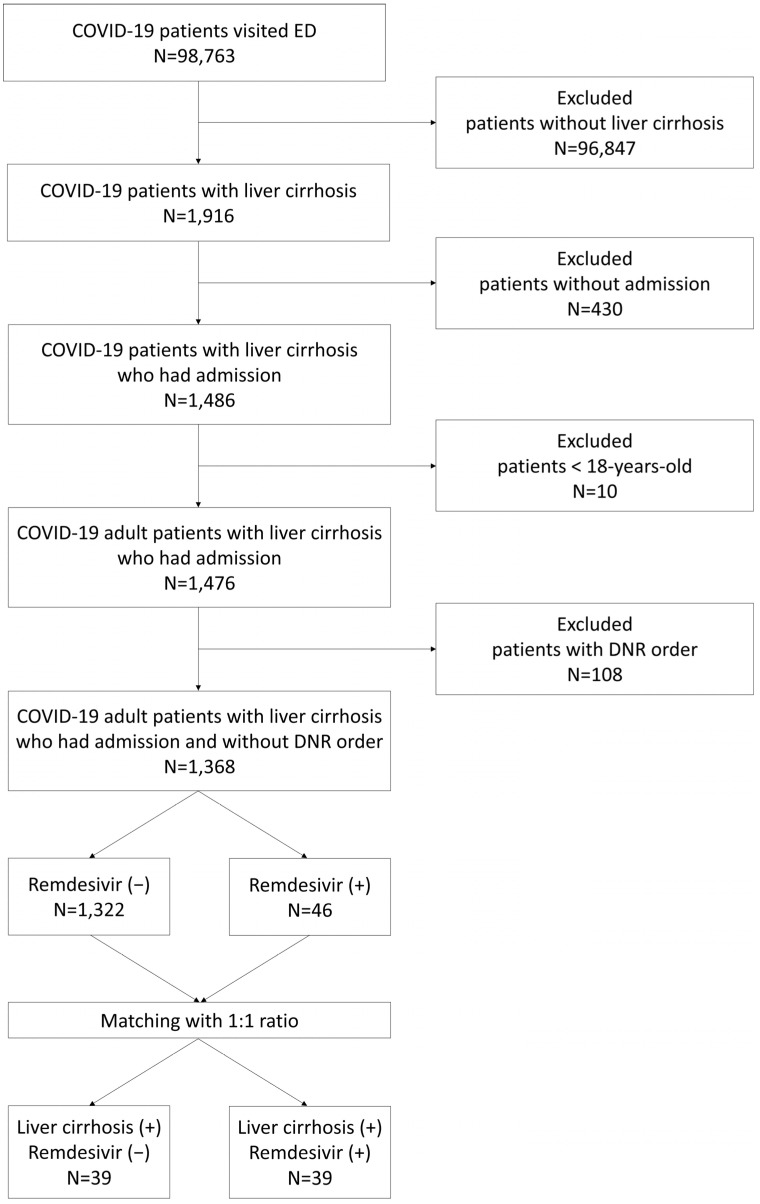
Flowchart depicting patient selection.

The CGRD included 98,763 patients who had received a COVID-19 diagnosis between April 2021 and September 2022 (Figure 1). Among them, 1916 patients had confirmed liver cirrhosis. After the exclusion of patients aged < 18 years, those not requiring hospitalization, and those with a DNR order, 1368 adult patients were included in this study. The patients were divided into two groups: remdesivir recipients and nonrecipients.

Table 1 presents the clinicodemographic characteristics of the remdesivir recipients and nonrecipients. Compared with the nonrecipients, the remdesivir recipients were older (66.5 vs. 62 years; *p* = 0.042) and had higher rates of requiring oxygen therapy (56.52% vs. 32.22%; *p* = 0.001) and concomitant dexamethasone (67.39% vs. 4.01%; *p* < 0.001). The mean score on the WHO ordinal scale was higher for the remdesivir recipients than for the nonrecipients (4 vs. 3; *p* = 0.017). However, no significant difference in the MELD score was observed between the remdesivir recipients and nonrecipients (14.41 vs. 14.48; *p* = 0.901). Moreover, no between-group difference was observed in the inflammation risk categories (*p* = 0.863).

The common side effects of remdesivir are listed in Table 2. Compared to nonrecipients, recipients of remdesivir showed lower heart rates (83 vs. 96 bpm; *p* < 0.001) and hemoglobin levels (9.5 and 10.2 g/dL; *p* = 0.003). However, there were no significant differences between remdesivir recipients and nonrecipients in terms of glucose levels (198.5 vs. 198.5 mg/dL; *p* = 0.795), bilirubin (0.6 vs. 0.9 mg/dL; *p* = 0.640), or alanine aminotransferase (ALT) (34 vs. 28.5 U/L; *p* = 0.242).

Other variables related to liver function, including international normalized ratio (INR) (1.3 vs. 1.3; *p* = 0.128), ammonia levels (90 vs. 78 μg/dL; *p* = 0.184), and albumin (3.18 vs. 2.87 g/dL; *p* = 0.417), showed no significant differences between patient groups. Renal functions indicators included creatinine (0.95 vs. 1 mg/dL; *p* = 0.149) and blood urea nitrogen (BUN) (29.8 vs. 19 mg/dL; *p* = 0.019), with only BUN levels showing elevation in remdesivir recipients.

The patients stratified by MELD score (≥14 vs. <14) were compared for adverse effects of remdesivir use (Supplementary Table S1) and no significant difference was found.

The remdesivir recipients and nonrecipients were compared after propensity score matching by sex, age, vital signs, oxygen use, and dexamethasone use (n = 39 per group; Table 3). Compared with nonrecipients, remdesivir recipients had reduced hospitalization duration (9 vs. 13.5 days; *p* = 0.059), intubation rates (12.82% vs. 30.77%; *p* = 0.100), and mortality rates (5.13% vs. 17.95%; *p* = 0.986), but the differences were nonsignificant.

## 4. Discussion

To evaluate the effectiveness of remdesivir in COVID-19 patients with liver cirrhosis, we adjusted the statistical model for potential confounders and performed propensity score matching based on age, sex, initial respiratory status, and liver cirrhosis severity by using the MELD score. The differences between the remdesivir recipients and nonrecipients were not statistically significant in our study, so we could only suggest that remdesivir treatment might have improved outcomes in terms of hospitalization duration, intubation rate, ICU admission rate, and mortality rate, but the definite effectiveness could not be confirmed in our study. The results may be limited due to our small sample size.

Remdesivir can exert adverse effects on various organ systems. Among its adverse effects on the hepatic system are elevated serum levels of aspartate aminotransferase (AST), ALT, and bilirubin [16,23,41,42,43]. COVID-19 itself may cause liver dysfunction; however, COVID-19-induced elevation of biochemical parameters is transient and remains within mild-to-moderate levels [41]. Both may raise concerns about deteriorated liver function from using remdesivir in patients with COVID-19 and liver cirrhosis. However, the present study involving patients with COVID-19 and liver cirrhosis revealed no significant difference in liver function between remdesivir recipients and nonrecipients.

Among the adverse effects of remdesivir on the cardiovascular system, bradycardia is most prevalent. The mechanism underlying this adverse effect remains unknown. However, bradycardia is transient and does not increase the risk of mortality [33,44,45,46,47,48]. This condition was observed in our cohort, but it did not result in any fatal outcomes. Among the adverse effects of remdesivir on the hematological system is a reduction in hemoglobin levels [16,43]. Such a reduction was observed in our study, but it did not lead to any fatal outcomes. Notably, our study did not reveal any significant between-group difference in the incidence of hyperglycemia, the last known adverse effect of remdesivir [16]. In Kang et al., the adverse effects of remdesivir did not differ significantly between patients with hepatic impairment at baseline and those without it [43]; this finding aligns with ours. In summary, our study confirmed the safety of a 5-day regimen of remdesivir in patients with liver cirrhosis.

Our findings on the effectiveness and safety of remdesivir in COVID-19 patients with liver cirrhosis should also be interpreted in the context of other available treatments. While remdesivir has been widely used in hospitalized patients requiring oxygen support, novel oral antivirals, such as nirmatrelvir/ritonavir (Paxlovid) and molnupiravir, have shown promising results in reducing disease progression in high-risk patients [49]. Unlike remdesivir, these medications are administered orally, which may provide an advantage in outpatient settings.

Studies have shown that nirmatrelvir/ritonavir can reduce the risk of progression to severe COVID-19 and shorten the disease course [50,51]. Although it is metabolized by the liver, it is still considered safe in patients with mild to moderate liver impairment (Child–Pugh class A or B) [52]. However, its high potential for drug–drug interactions remains a major consideration in clinical prescribing [52,53]. As for molnupiravir, it has been shown to reduce the risk of hospitalization and death in mild disease and does not require dose adjustment in patients with liver impairment [54]. Among the three antiviral agents, remdesivir has been associated with the highest efficacy in preventing hospitalization in high-risk symptomatic COVID-19 patients [55].

In Taiwan, before mid-May 2021, COVID-19 did not cause a major outbreak, and the predominant strain was the original virus strain. After mid-May 2021, a nationwide outbreak occurred, with the Alpha variant (B.1.1.7) becoming the predominant strain. In July 2021, the Delta variant (B.1.617.2) spread globally and eventually replaced Alpha as the predominant strain in Taiwan in August, but it did not cause a nationwide outbreak as Alpha had. Starting in early 2022, the Omicron variant (B.1.1.529) became the predominant strain, later being replaced by the Omicron BA.2 subvariant in late March 2022 [56]. The definite strains were not confirmed in our included patients, but Omicron strains were considered the predominant strain, and some Delta strains may exist in our patients. Although some mutations resistant to remdesivir were noted in some studies, most variants and subvariants of SARS-CoV-2 have remained susceptible to remdesivir [55].

### Limitations

This study has some limitations. First, as this was a retrospective study, our analysis was limited to previously collected data. Additionally, due to the nature of our database, only structured data could be collected. Information recorded in clinical notes was not categorized into predefined fields, such as the Child–Pugh score, ascites, consciousness level, and variceal bleeding. Second, the sample size for each group was small after propensity score matching. Additionally, to avoid a potential bias from confounders, we excluded patients with a DNR order, which further reduced the sample size. Moreover, this was a single-country, multicenter study; the study design might have introduced a selection bias and may have limited the applicability of our findings to other regions or countries. Finally, factors such as the cycle threshold value in polymerase chain reaction and vaccination status were not explored because of the unavailability of such data; this reduced the comprehensiveness of our results.

## 5. Conclusions

Our study provides insights into the safety and effectiveness of a 5-day remdesivir regimen in patients with COVID-19 and liver cirrhosis. Some adverse effects, such as bradycardia and anemia, were observed but did not result in any fatal consequences. Remdesivir recipients tended to have better outcomes in terms of hospitalization duration, intubation rate, and mortality, while these improvements were not statistically significant compared to those in nonrecipients, which may be associated with small sample size. In summary, remdesivir appears to be safe for use in patients with COVID-19 and liver cirrhosis and may have potential effectiveness. Further research is still necessary to confirm its efficacy.

## Figures and Tables

**Table 1:** Clinicodemographic characteristics of remdesivir recipients and nonrecipients among patients with COVID-19 and liver cirrhosis.

	Non-RDSV (N = 1322)	RDSV (N = 46)	*p*-Value
Age, years, median (IQR)	62 (53–72)	66.5 (56–82)	**0.042**
Gender, male, n (%)	924 (69.89)	32 (69.57)	1
Dexamethasone use, n (%)	53 (4.01)	31 (67.39)	**<0.001**
Oxygen use, n (%)	426 (32.22)	26 (56.52)	**0.001**
Initial vital signs			
BT, °C, median (IQR)	36.5 (36–37.1)	37.4 (36.8–38.2)	**<0.001**
SBP, mmHg, median (IQR)	134.5 (115–155)	132.5 (110.5–146.5)	0.381
DBP, mmHg, median (IQR)	75 (65–87)	76.5 (70–85)	0.716
RR, breaths per minute, median (IQR)	17 (16–19)	18 (16–20)	**0.001**
HR, bpm, median (IQR)	96 (83–110)	101 (91–112)	**0.032**
SpO_2_, %, median (IQR)	97 (95–98)	96 (93–98)	0.121
Initial laboratory data			
WBC, 1000/uL, median (IQR)	7.1 (4.9–10.4)	6.5 (4.5–8.4)	0.164
Hb, g/dL, median (IQR)	10.2 (8.6–12)	11.5 (10.2–12.2)	**0.005**
Creatinine, mg/dL, median (IQR)	1 (0.71–1.57)	1 (0.7–1.85)	0.953
BUN, mg/dL, median (IQR)	23.5 (15.3–39.8)	19 (14.7–38.2)	0.480
ALT, U/L, median (IQR)	32 (20–54)	28.5 (17–38)	**0.049**
Bilirubin, mg/dL, median (IQR)	1.6 (0.8–3.3)	0.9 (0.55–1.9)	**0.015**
Ammonia, μg/dL, median (IQR)	102.5 (61–160)	78 (46–141)	0.389
Albumin, g/dL, median (IQR)	3.09 (2.69–3.5)	2.87 (2–3.25)	0.096
CRP, mg/L, median (IQR)	22.52 (7.36–70.39)	20.28 (8.74–99.36)	0.521
Glucose, mg/dL, median (IQR)	120 (98–165)	198.5 (127–282)	**0.012**
Na, mEq/L, median (IQR)	135 (131–138)	136 (132–138)	0.878
K, mEq/L, median (IQR)	4 (3.5–4.4)	4.1 (3.5–4.4)	0.646
Troponin I, ng/mL, median (IQR)	0.02 (0.01–0.06)	0.03 (0.01–0.07)	0.542
D-dimer, ng/mL, median (IQR)	2606 (1174–5572)	365 (365–365)	0.153
Prothrombin time, INR, median (IQR)	1.3 (1.2–1.5)	1.3 (1.1–1.4)	0.4660
Prognosis			
LOS, days, median (IQR)	10 (6–18)	9 (6–14)	0.140
Intubation, n (%)	191 (14.45)	6 (13.04)	0.958
ICU admission, n (%)	115 (86.98)	<3	0.984
Mortality, n (%)	76(5.75)	3 (6.52)	0.503
Underlying diseases			
Cardiovascular disease, n (%)	33 (2.5)	0 (0)	1
Hypertension, n (%)	648 (49.02)	28 (60.87)	0.153
Congestive heart failure, n (%)	151 (11.42)	10 (21.74)	0.057
Cerebrovascular disease, n (%)	198 (14.98)	10 (21.74)	0.295
Chronic pulmonary disease, n (%)	198 (14.98)	13 (28.26)	**0.025**
Diabetes mellitus, n (%)	595 (45.01)	23 (50)	0.604
Malignancy, n (%)	687 (51.97)	25 (54.35)	0.867
Renal disease, n (%)	384 (29.05)	13 (28.26)	1
MELD score, median (IQR)	14.48 (10.68–19.73)	14.41 (11.6–21.46)	0.901
WHO ordinal scale, median (IQR)	3 (3–4)	4 (3–4)	**0.017**
Inflammation risk categories, n (%)			0.863
H	494 (37.37)	19 (41.30)	
I	305 (23.07)	10 (21.74)	
L	523 (39.56)	17 (36.96)	

*p*-values < 0.05 are highlighted in bold. Abbreviations: RDSV: remdesivir; BT: body temperature; SBP: systolic blood pressure; DBP: diastolic blood pressure; RR: respiratory rate; HR: heart rate; bpm: beats per minute; WBC: white blood cell; Hb: hemoglobin; BUN: blood urea nitrogen; ALT: alanine transaminase; INR: International Normalized Ratio; LOS: length of stay; ICU: intensive care unit; MELD: model for end-stage liver disease; WHO: World Health Organization; IQR, interquartile range.

**Table 2:** Adverse effects of remdesivir use.

	Before RDSV	After RDSV	*p*-Value
HR, bpm, median (IQR)	96 (83–110)	83 (72–96)	**<0.001**
Hb, g/dL, median (IQR)	10.2 (8.6–12)	9.5 (8.4–11.1)	**0.003**
Glucose, mg/dL, median (IQR)	198.5 (127–282)	198.5 (114–326)	0.795
Bilirubin, mg/dL, median (IQR)	0.9 (0.55–1.9)	0.6 (0.35–1.55)	0.640
ALT, U/L, median (IQR)	28.5 (17–38)	34 (18–58)	0.242
Prothrombin Time, INR, median (IQR)	1.3 (1.1–1.4)	1.3 (1.2–1.5)	0.128
Ammonia, μg/dL, median (IQR)	78 (46–141)	90 (63–156)	0.184
Albumin, g/dL, median (IQR)	2.87 (2–3.25)	3.18 (2.65–3.72)	0.417
Creatinine, mg/dL, median (IQR)	1 (0.7–1.85)	0.95 (0.66–2.39)	0.149
BUN, mg/dL, median (IQR)	19 (14.7–38.2)	29.8 (18.6–56.9)	**0.019**

*p*-values < 0.05 are highlighted in bold. Abbreviation: RDSV: remdesivir; HR: heart rate; bpm: beats per minute; Hb: hemoglobin; ALT: alanine transaminase; BUN: blood urea nitrogen; INR: international normalized ratio; IQR, interquartile range.

**Table 3:** Clinicodemographic characteristics of remdesivir recipients and nonrecipients after propensity score matching.

	Non-RDSV (N = 39)	RDSV (N = 39)	*p*-Value
Characteristics			
Age, years, median (IQR)	67 (57–75)	64 (53–81)	0.853
Gender, male, N (%)	26 (66.67)	26 (66.67)	1
Dexamethasone use, n (%)	25 (64.1)	25 (64.1)	1
Oxygen use, n (%)	15 (38.46)	22 (56.41)	0.174
MELD score, median (IQR)	13.31 (8.73–16.81)	14.47 (11.57–22.75)	0.497
MELD-Na score, median (IQR)	17.4 (13.47–22.3)	16.79 (15.06–23.32)	0.810
WHO ordinal scale, median (IQR)	3 (3–4)	4 (3–4)	0.644
X-ray score			0.416
0	10	4	
1	<3	4	
2	20	23	
3	<3	<3	
4	<3	<3	
Outcome			
LOS, days, median (IQR)	13.5 (6–22)	9 (6–14)	0.059
Intubation, n (%)	12 (30.77)	5 (12.82)	0.100
ICU admission, n (%)	4 (10.26%)	<3	0.973
Mortality, n (%)	7 (17.95)	2 (5.13)	0.986

Abbreviations: RDSV: remdesivir; LOS: length of stay; ICU: intensive care unit; MELD: model for end-stage liver disease; WHO: World Health Organization; IQR: interquartile range.

## Data Availability

The data that support the findings of this study are available from Linkou Chang Gung Memorial Hospital, but restrictions may apply to the availability of these data, which were approved by the individual hospital IRB for the current study, and such they are not publicly available. However, processed datasets can be requested and made available from the authors with the permission of Linkou Chang Gung Memorial Hospital.

## Supplementary Materials

The following supporting information can be downloaded at: https://www.mdpi.com/article/10.3390/life15040512/s1. Table S1: Adverse effects of remdesivir use in patients of MELD score ≥ 14 and <14.
