# Supplementary material for: Effectiveness and Safety of Remdesivir for the Treatment of COVID-19 Patients with Liver Cirrhosis: A Retrospective Cohort Study
Source: Life (Basel). 2025 Mar 21;15(4):512. doi: 10.3390/life15040512 (PMC12028772; doi:10.3390/life15040512)
Supplement: Supplementary file 1 [file life-15-00512-s001.zip › life-3491447-supplementary.pdf]

Supplementary table

Supplementary Table S1-1. Adverse effects of remdesivir use in patients of MELD score ≥ 14 (n=11)

	Before RDSV	After RDSV[#]	p-value
HR, bpm, median (IQR)	92.5(84-105)	91(73-119)	**0.046**
Hb, g/dL, median (IQR)	10.2(9.2-11.9)	10.8(9-11.7)	0.261
Glucose, mg/dL, median (IQR)	249(198.5-390)	310.5(198.5-446)	0.716
Bilirubin, mg/dL, median (IQR)	2.7(0.8-3.3)	1.4(0.5-5.8)	0.380
ALT, U/L, median (IQR)	35.5(14-45)	44.5(12-78)	0.335
Prothrombin Time, INR, median (IQR)	1.3(1.2-1.7)	1.5(1.4-2.3)	0.247
Ammonia, μg/dL, median (IQR)	133(36-241)	123(81.5-158.5)	0.136
Albumin, g/dL, median (IQR)	2.56(2.26-3.29)	2.52(2.35-3.05)	0.834
Creatinine, mg/dL, median (IQR)	2.18(1.16-3.95)	2.31(0.86-3.02)	0.629
BUN, mg/dL, median (IQR)	50.05(31.6-61.35)	72.1(18.6-92.5)	**0.036**

P-values < 0.05 are highlighted in bold. **Abbreviation:** RDSV: remdesivir; HR: heart rate; bpm: beats per minute; Hb: hemoglobin; ALT: alanine transaminase; BUN: blood urea nitrogen; INR: International Normalized Ratio; IQR, interquartile range.

Supplementary Table S1-2. Adverse effects of remdesivir use in patients of MELD score <14 (n=35)

	Before RDSV	After RDSV#	p-value
HR, bpm, median (IQR)	102(95-112)	88(84-96)	**<0.001**
Hb, g/dL, median (IQR)	11.55(10.4-12.5)	10.25(9.7-11.8)	**0.006**
Glucose, mg/dL, median (IQR)	140(113-265)	140(92.5-265)	0.351
Bilirubin, mg/dL, median (IQR)	0.7(0.5-1.2)	0.5(0.3-0.8)	0.057
ALT, U/L, median (IQR)	27(17.5-37)	34(18-43)	0.476
Prothrombin Time, INR, median (IQR)	1.3(1.1-1.35)	1.3(1.1-1.4)	0.062
Ammonia, μg/dL, median (IQR)	74(46-103)	86(63-130)	0.102
Albumin, g/dL, median (IQR)	3.02(1.74-3.21)	3.39(2.79-3.85)	0.424
Creatinine, mg/dL, median (IQR)	0.85(0.69-1.05)	0.78(0.66-1.08)	0.177
BUN, mg/dL, median (IQR)	17.2(14.6-22.8)	27.55(17.2-48.9)	0.256

P-values < 0.05 are highlighted in bold. **Abbreviation:** RDSV: remdesivir; HR: heart rate; bpm: beats per minute; Hb: hemoglobin; ALT: alanine transaminase; BUN: blood urea nitrogen; INR: International Normalized Ratio; IQR, interquartile range.
